# Twenty Intracranial Skull Base Variations in the Same Specimen

**DOI:** 10.7759/cureus.40096

**Published:** 2023-06-07

**Authors:** Mitchell Couldwell, Arada Chaiyamoon, Juan J Cardona, Joe Iwanaga, David Ezra, Athikhun Suwannakhan, Aaron S Dumont, R. Shane Tubbs

**Affiliations:** 1 Department of Neurosurgery, Tulane University School of Medicine, New Orleans, USA; 2 Department of Anatomy, Faculty of Medicine, Khon Kaen University, Khon Kaen, THA; 3 School of Nursing Science, The Academic College of Tel Aviv-Yaffo, Yaffo, ISR; 4 Department of Anatomy, Faculty of Science, Mahidol University, Bangkok, THA; 5 Department of Neurology, Tulane University School of Medicine, New Orleans, USA; 6 Department of Anatomical Sciences, St. George’s University, St. George’s, GRD; 7 Department of Surgery, Tulane University School of Medicine, New Orleans, USA; 8 Department of Structural and Cellular Biology, Tulane University School of Medicine, New Orleans, USA; 9 Department of Neurosurgery, Ochsner Neuroscience Institute, Ochsner Health System, New Orleans, USA

**Keywords:** cranium, intracranial, cadaver, skull, variations, anatomy

## Abstract

Anatomists and clinicians often encounter single bony anatomical variations in dry skulls and on imaging. However, a constellation of 20 such variants some that, to our knowledge, have not been previously described is noteworthy. Here, we describe an adult skull with multiple bony variations, and these are detailed and discussed. These included clival canals, an interclinoid bar with resultant foramen at the uppermost aspect of the clivus, middle clinoid process, posterior petroclinoid ligament, pterygoalar plate, septated hypoglossal canal, foramen through the anterior clinoid process, septated foramen ovale, shortened superior orbital fissure, and crista muscularis. Knowledge of individual differences in the structure of the skull may be of use to both anatomists and clinicians in the treatment of intracranial procedures as well as cranial imaging studies. Taken together, such a unique specimen is of archival value.

## Introduction

The skull is a complex bony structure that supports and encloses the brain and its many variations have been studied for many years and have drawn interest from both clinicians and anatomists alike [1-26]. Differences among individuals and elucidations of variation in bony structures of the cranium may be important considerations for the clinician during imaging studies, diagnosis, and treatment of neurological conditions.

For example, anatomical variations seen on imaging of the skull must be distinguished from pathology and variations found at operation of the skull must be well understood in order to avoid iatrogenic complications.

The skull is composed of both the neurocranium, which consists of the bones which support and enclose the brain and are derived from mesenchyme, and the viscerocranium, which consists of bones of the facial skeleton that are derived from the first three pharyngeal arches [3,4]. Development of the skull and head begins with the formation of the neural crest which constitutes the embryological origin of the connective and skeletal tissues of the skull. Migration of neural crest cells around the early pharynx leads to the formation of five sets of pharyngeal arches each of which contain ectoderm, mesenchyme-derived neural crest cells, and endoderm [5]. The first pharyngeal arch forms the malleus and incus. The second pharyngeal arch contributes to the stapes, the styloid process of the temporal bone, and the lesser horn of the hyoid. The third pharyngeal arch contributes to the body of the hyoid as well as its greater horn [3].

The neurocranium can be further divided into the chondrocranium and the calvaria, which refer to bones of the cranial base, and the bones which surround the brain, respectively. The chondrocranium forms the base of the skull including the sphenoid, the ethmoid, and the occipital bones. The hypophysial cartilage, which constitutes the body of the sphenoid, forms at the edge of the developing pituitary gland and then fuses, meanwhile, the wing of the sphenoid is formed by the ala orbitalis. The base of the occipital bone is formed by a mass of tissue derived from the parachordal cartilage which forms around the cranial end of the notochord which then fuses with cartilage derived from the occipital somites. Later in development, this mass grows around the cranial end of the spinal cord to form the boundaries of the foramen magnum. Otic capsules grow around the otic vesicles in the early inner ear and form the mastoid and petrous parts of the temporal bone [3].

The calvaria forms through membranous ossification of the mesenchyme at the top and sides of the brain, and dense connective tissue forms fibrous joints which will become the sutures of the skull and these allow uniform growth of the calvaria as the brain grows [5]. The viscerocranium is formed through membranous ossification in the maxillary and mandibular prominences of the first pharyngeal arch. The maxillary prominence gives rise to the maxillary bones, zygomatic bones, as well as the squamous part of the temporal bones which will become part of the neurocranium. The mandibular prominence forms the mandible through membranous ossification although some endochondral ossification occurs in the mandibular condyle as well as the midsagittal plane of the chin [3]. The mechanism of cranial foramina formation within the endochondral ossified skull base is thought to involve cellular clearing and adaptation for the growth of nerves and blood vessels contained within these spaces [6].

Variations in cranial anatomy have been widely discussed, usually in isolation. Here, the authors present an example of a specimen with the following main variations: multiple clival canals, pterygoid spinous bony bars resulting in the additional foramina for contents of foramen spinosum and foramen ovale, the middle clinoid process and resultant carotico-clinoid foramen, an ossified petroclinoid ligament, a septated hypoglossal canal, and a septated left jugular foramen. Three additional skull variations that, to our knowledge, have not been previously discussed in the literature included a foramen through the anterior clinoid process, a very shortened superior orbital fissure, and a foramen located at the dorsum sellae [7].

## Case presentation

**Figure 1:**
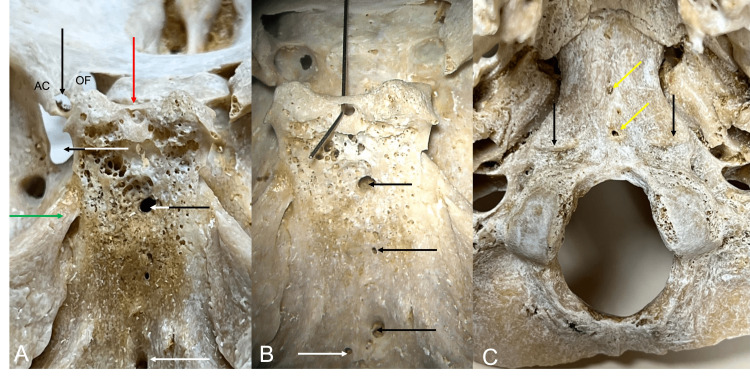
Left: Note the left anterior clinoid process (AC) with unusual foramen passing through it (black arrow). The optic foramen is seen just medial to this as well as the bony bar (red arrow) between the posterior clinoid processes with resultant foramen. The shortened left superior orbital fissure (black/white arrow) and ossified petroclinoid ligament (green arrow) are seen. Two of the large clival canals are seen at the white/black arrow and white arrow. Middle: A metal wire is inserted into the foramen formed by the interclinoid bony bar. Large and small clival canals are seen at the arrows. Right: Inferior view noting the crista muscularis (black arrows), and two clival canals that have pierced the bone (yellow arrows).

**Figure 2:**
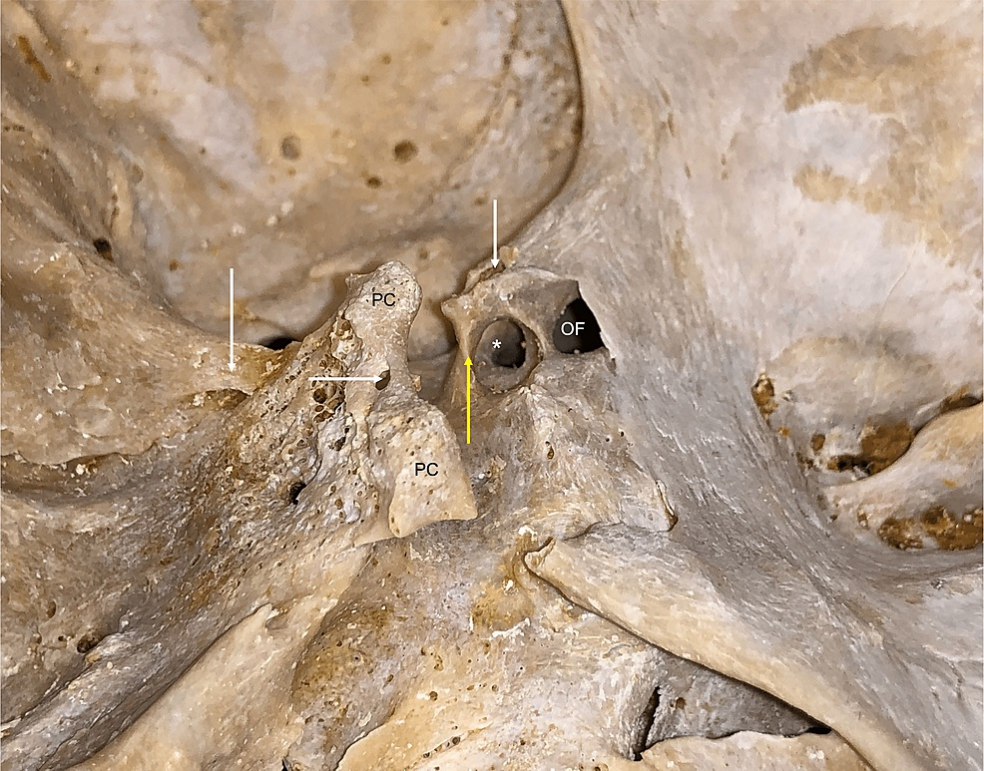
Lateral view noting the ossified posterior petroclinoid ligament (left vertical white arrow), foramen (horizontal white arrow) formed by the interclinoid bony bar, middle clinoid process (yellow arrow) with resultant caroticoclinoid foramen (*). A foramen is noted traveling through the left anterior clinoid process (right vertical white arrow). Note the optic foramen (OF).

**Figure 3:**
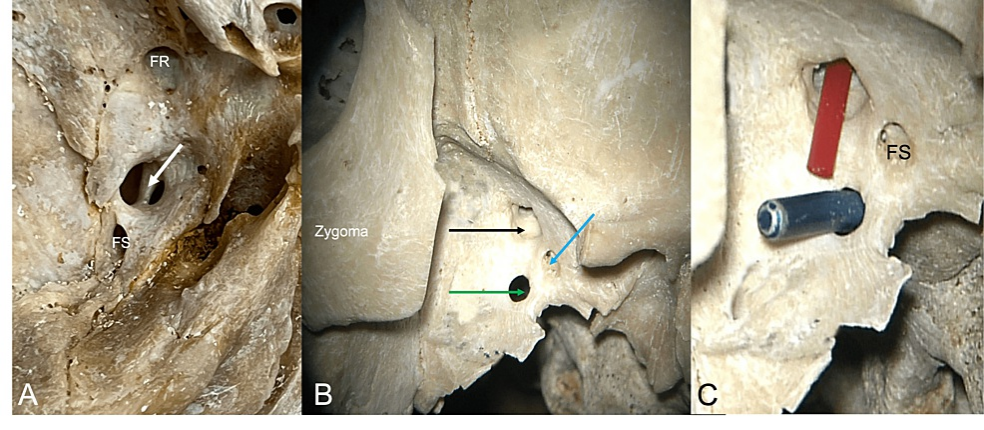
Left: Floor of the left middle cranial fossa noting the foramen rotundum (FR), foramen spinosum (FS), and septated foramen ovale (arrow). Middle: Following removal of the zygomatic arch, the large plate of bone extending off of the pterygoid process is easily seen. Right: red tubing is inserted through the lateral part of the septated foramen ovale and a blue wire is placed into the unnamed foramen. Note the foramen spinosum (FS) and foramen ovale are both integrated into this bony sheet of bone. Note the foramen ovale (black arrow), foramen spinosum (blue arrow), and unnamed foramen (green arrow).

**Figure 4:**
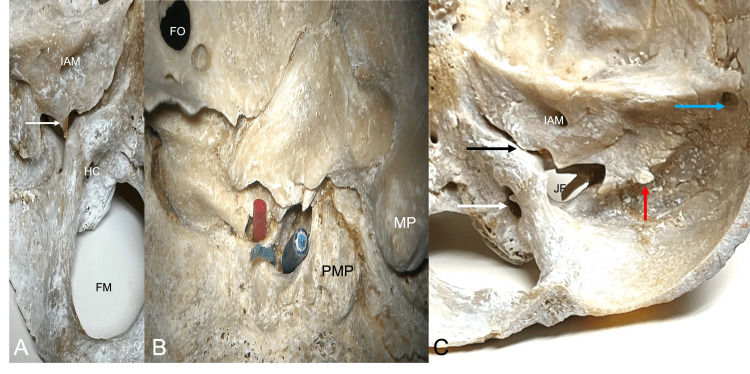
Left: View into the posterior cranial fossa. Middle: Inferior view of the left skull base noting the foramen ovale (FO), mastoid process (MP), paramastoid process (PMP), and the external septation of the jugular foramen with three compartments as shown by the red, green, and blue wires. Right: View of the right posterior cranial fossa noting the internal acoustic meatus (IAM), jugular foramen (JF), and septated hypoglossal canal (white arrow). Note the internal acoustic meatus (IAM), hypoglossal canal (HC), foramen magnum (FM), and the more internal septation within the left jugular foramen (arrow). Also note the bony canal (black arrow) formed for the inferior petrosal sinus, osteophyte (red arrow) protruding toward the groove for the sigmoid sinus, and large granular foveola (blue arrow) at the junction of the grooves for the transverse and sigmoid sinuses.

During the routine evaluation of an older adult skull derived from a female skeleton, multiple anatomical variations were identified at the skull base. In the midline, multiple clival canals were seen ranging in size from 0.85 to 2.23 mm in diameter (Figure 1). Each of these communicated with the diploic space of the clivus and only two of these pierced the bone to lie on its inferior surface (Figure 1). Between the left and right posterior clinoid processes, an interclinoid bony bar (Figure 1) was found which resulted in a midline foramen (1.47 mm) between these two processes. A left middle clinoid process with a resultant carotico-clinoid foramen (Figure 2) was observed, and a foramen was seen traveling through the anterior clinoid process This foramen through the anterior clinoid process ended at the apex of the orbit (Figures 1-3). A large left-sided ossified posterior petroclinoid ligament (8.5 mm x 4.1 mm) was seen (Figures 1, 2). The left floor of the foramen ovale was septated (Figure 3) with the medial portion of this septated foramen connecting medially to a large and fused pterygoalar plate of bone that itself was penetrated by an additional foramen (3.4 mm in diameter) (Figure 3). The ipsilateral foramen spinosum’s external opening and spine were fused into this plate of bone (Figure 3). The lateral portion of the septated foramen ovale is connected to the space lateral to the large fused pterygoalar plate of bone (Figure 3). A septated right hypoglossal canal and a septated left jugular foramen with one internal and two external septations were identified (Figure 4). The left superior orbital fissure was shortened (9.6 mm in length) compared to the right side (17.1 mm in length) (Figure 1). There was also a right-sided inferior petrosal sinus canal instead of an inferior petrosal sinus groove (Figure 4). Other remarkable structures included a prominent granular foveola of the left sigmoid sulcus and a bony protuberance (3.7 x 3.4 mm) of the petrous part of the temporal bone protruding posteriorly into the sigmoid sulcus (Figure 4). A crista muscularis was observed on left and right sides as well as small bilateral paramastoid processes (Figures 1, 4). The authors state that every effort was made to follow all local and international ethical guidelines and laws that pertain to the use of human cadaveric donors in anatomical research [27].

## Discussion

Variations in the clivus have been discussed in the literature and multiple instances of both vertical and horizontal canals have been reported. These variations include a fossa navicularis magna (FNM) and canalis basilaris medianus (CBM). The FNM or also known as a pharyngeal fossa or phyaryngeal fovela is a notch-like variant in the midline of the occipital part of the clivus. The CBM is identified as a variant corticated canal in the basiocciput. It is classified into six types depending on its morphology [9]. Lang and Samii identified clival canals in 2% of specimens, while Nayak et al., as well as Jalsovec and Vinter, had similar findings [1,9,10]. Most recently, Tubbs et al. reported an overall prevalence of clival canals of 5.44% in a study consisting of 100 dry skulls and 47 embalmed cadavers [11]. Five clival foramina were found in the current case (Figure 1).

Anatomical variations in the foramen ovale and foramen spinosum have been previously described. Tubbs et al. described a study in which 154 dry adult skulls were analyzed for ossification of the pterygospinous and pterygoalar ligaments and two completely ossified examples of both were found. One complete ossification of each was also found resulting in the foramen ovale dividing into two compartments and this was also seen in the present specimen (Figure 3) [12]. Khan et al. reported a case of a dry skull with a bony bar separating the foramen spinosum into anterior and posterior compartments [13]. Ray et al. reported that in a study of 35 skulls, the foramen ovale was divided into two compartments by a bony spur in two instances, once on the right side and once on the left [14]. Lastly, Reymond et al. reported in a study of 100 skulls that the foramen ovale was split into two or three compartments in 4.5% of cases [15]. Our case is interesting in that a broad plate of bone was formed below the foramen ovale and this allowed medial and lateral compartments within the foramen ovale to be segregated inferiorly. Moreover, a large foramen in this plate of bone was identified (Figure 3).

The middle clinoid process forming a foramen with the anterior clinoid process has been described multiple times in the literature and is often referred to as the carotico-clinoid foramen (Figure 2). Ozdoğmuş et al. studied 50 autopsy specimens and examined the clinoid processes bilaterally and reported complete foramina in 27 sides and incomplete foramina in 18 sides of specimens [16]. Furthermore, Erturk et al. reported findings of both complete and incomplete foramina in a study of 119 dry skulls and 52 cadaveric heads and reported complete foramina in approximately 4% of the specimens and incomplete in approximately 15% [17]. The interclinoid bony bar with resultant foramen (Figure 1) appears to be unique in our bony specimen.

Ossification of the petroclinoid ligament has been reported multiple times (Figure 2). Wysiadecki et al. described a cadaveric case in which bilateral ossification of the posterior petroclinoid ligament was observed in the dissection of a 76-year-old female cadaver [18]. Inal et al. used multi-slice computed tomography to review 130 skull bases and reported findings of complete ossification of 5.2% on the right side, 4.6% on the left side, and partial ossification of 26.6% on the left side and 29.5% on the right [19].

Multiple large studies of septation and duplication of hypoglossal canals have been described previously (Figure 4). Kaur et al. discussed the findings of double hypoglossal canals in a study in a study of 57 male and 33 female dry skulls originating from northern India in which 3.5% of male skulls presented with unilateral double hypoglossal canals on the right, and 7% on the left, and 5.3% bilaterally, while the female skulls presented with 6.1% unilaterally on both the left and the right, and 3% bilaterally [20]. Kanda et al. described a study of 590 patients who underwent computed tomographic angiography and found that double hypoglossal canals were present unilaterally in 16.9% of patients and bilaterally in 2.2% [21]. Furthermore, Tubbs et al. discussed a single case in a skull that presented with bony septation of both hypoglossal canals [22].

Descriptions of septation of the jugular foramen were discussed by Hatiboğlu et al. in a study of 300 dry skulls [23]. The authors noted septation of the right jugular foramen in 5.6% of specimens and septation of the left side in 4.6% of specimens. Those with bilateral or multiple septations such as seen in our specimen (Figure 4) were not reported. Kaur et al. discussed a study of 57 male and 33 female dry skulls in which the jugular foramen bridging was present in male skulls on the right side in 7% of specimens, 5.2% on the left side, and bilaterally in 1.7% of cases [20]. In female skulls, the incidence was 12.1% on the left side, 6.1% on the right side, and 3% bilaterally [20]. Gupta et al. studied 50 dry skulls and found that complete septation was found in 44% on the right side and 42% on the left side [24]. We recently published an anatomical study where the different septations of the jugular foramen were classified [2,25].

Hofmann and Prescher have previously mentioned the crista muscularis (Figure 1) and believed it was due to the attachment of the rectus capitis anterior muscle [26]. A granular foveola along the midline of the superior sagittal sinus groove is common. However, such depressions due to large arachnoid granulations are uncommon along the sulci for the transverse and sigmoid sinuses [28].

Declaration

The authors sincerely thank those who donated their bodies to science so that anatomical research could be performed. Results from such research can potentially increase mankind’s overall knowledge that can improve patient care. Therefore, these donors and their families deserve our highest gratitude [29].

## Conclusions

Knowledge of individual differences in the structure of the skull may be of use by both anatomists and clinicians. Single bony variations are commonly seen and in isolation, most of the variations reported in the present case are not noteworthy. However, taken together, such a unique specimen is of archival value. Additionally, some of the anatomical variations demonstrated in the current case report are novel and have, to our knowledge, not been previously reported e.g., foramen traversing the anterior clinoid process.
